# Cannabis use in early ADHD, a 3-years follow-up study in relations to clinical characteristics

**DOI:** 10.1192/j.eurpsy.2022.222

**Published:** 2022-09-01

**Authors:** L.R. Kvitland, H. Moa, J. Schei, S. Lydersen, P. Thomsen, I. Mundal

**Affiliations:** 1 Regional Centre for Child and Youth Mental Health and Child Welfare, Department Of Mental Health, Norwegian University Of Science And Technology, Trondheim, Norway; 2 Orkanger BUP, Bup Clinic, St. Olavs Hospital, Orkanger, Norway; 3 Aarhus University Hospital Psychiatry, Department Of Child & Adolescent Psychiatry, Aarhus N, Denmark

**Keywords:** Cannabis, out-patient, adhd

## Abstract

**Introduction:**

ADHD is known to increase the risk of substance use, and is associated with lower degrees of education, criminal behavior and neuropsychic difficulties. Previous research is limited by small samples, variable findings, and short follow-up time. Earlier research tends to be limited to substance use above the threshold for abuse or dependency.

**Objectives:**

This study aims at looking at the effects of cannabis use both over and under threshold for abuse or dependency in relations to clinical characteristics over a 3-year follow-up period.

**Methods:**

At follow up a total of 203 patients were diagnosed with ADHD either as primary of as secondary diagnosis, of those 57 (28,1%) had lifetime use of cannabis (LUC), mean age at inclusion was 15 and half years old and 40% were of female sex. SPSS (v.29) were used to perform independent sample t-tests to test for effects and Hierarchical block-wise regressions were done to check for confounding variables.

**Results:**

Lifetime cannabis use was associated with lower global functioning (p=0.000), increased risk of suicidal ideation (p=0.007), more suicide attempts (p=0.049), more self-reported symptoms (p=0.001), more school drop-out (p=0.000) and with psychotic features (p=0.024). Even after testing for know confounders such as female sex and age LUC explained significant variance.

**Conclusions:**

LUC is associated with increased functional and clinical characteristics. The findings are discussed in relationship with clinical practice and limitations of the study.

**Disclosure:**

No significant relationships.

